# Don’t Miss the Blind Spots: Incidental Detection of Choroidal Melanoma During Primary Care Evaluation of Visual Field Loss

**DOI:** 10.7759/cureus.109650

**Published:** 2026-05-25

**Authors:** Omar J Bishr, Adityanarayan Rao, Aruna Thomas, Saji Packal

**Affiliations:** 1 Internal Medicine, University of Florida, Gainesville, USA; 2 College of Agricultural and Life Sciences, University of Florida, Gainesville, USA; 3 Internal Medicine, Malcom Randall Department of Veterans Affairs Medical Center, Gainesville, USA

**Keywords:** case report, choroidal malignant melanoma, intraocular malignancy, ocular melanoma, uveal melanoma

## Abstract

Identifying choroidal melanoma in the primary care setting represents a diagnostic challenge. We present a rare case of choroidal melanoma in a patient who presented with progressive superolateral quadrantanopia. A CT of the head identified an intraocular hyperdensity within the left globe. Fundoscopy confirmed a classic dome-shaped choroidal melanoma. Gene expression profiling demonstrated a class 1b uveal melanoma with PRAME positivity and mutations in GNAQ and SF3B1. Staging scans were negative for metastatic disease. Vision improved following four sessions of proton beam therapy. This case highlights the importance of multidisciplinary involvement, multimodal imaging, and gene expression profiling in facilitating early diagnosis and treatment.

## Introduction

Choroidal malignant melanoma is a subtype of uveal melanoma and is the most common intraocular malignancy in adults. The disease predominantly affects the Caucasian population, with an incidence of roughly five cases per million annually in the United States [1]. Risk factors often include fair skin, light eye color, ocular melanocytosis, and BAP1 genetic mutations [2]. Uveal tract melanoma most commonly arises in the choroid (90%) but can also affect the ciliary body (6%) and iris (4%) [3]. The mortality rate of choroidal melanoma varies significantly between localized and metastatic disease. For localized choroidal melanoma, the five-year survival rate is typically 92-98% depending on treatment modality and tumor size [4,5]. Conversely, in metastatic disease, the median survival after proton therapy is approximately 1.25 years, with a five-year survival rate of only 6.0% [6]. This case highlights the importance of carefully evaluating ocular concerns as a primary care physician, as subtle physical examination findings can serve as early clues to underlying ocular cancers. Early recognition and diagnosis are vital to enable timely treatment and reduce the risk of metastasis. Unfortunately, detection in the primary care setting can be challenging due to asymptomatic or nonspecific presentations and a lack of specialized ophthalmic equipment or training. 

This article was previously presented as a poster at the 2025 Association of VA Hematology/Oncology Annual Meeting on September 12, 2025.

## Case presentation

A 57-year-old male patient with a past medical history significant for hypertension, hyperlipidemia, major depressive disorder, and alcohol use disorder presented to the primary care clinic for follow-up one month after an admission for voluntary alcohol detoxification. He reported a one-week history of progressive visual loss in the left eye, primarily affecting the left peripheral and central visual fields. He otherwise denied any flashes, floaters, photophobia, eye pain, or headaches. There was no history of trauma or similar symptoms in the past. He did not have any personal or family history of malignancy. 

His vital signs, including blood pressure, were within normal range. Physical examination was notable for a superior temporal quadrantanopia of the left eye, with preserved visual fields elsewhere. Extraocular movements were intact. Visual acuity was measured at 20/40 in the left eye and 20/30 in the right eye. His neurologic examination was otherwise unremarkable, without any focal deficits. 

A CT of the brain without contrast was urgently obtained due to concern for cerebrovascular accident (CVA). The CT did not demonstrate acute ischemic changes but did show a lentiform intraocular hyperdensity along the posteromedial aspect of the left globe, extending toward the optic disc, which was concerning for retinal detachment. 

**Figure 1:**
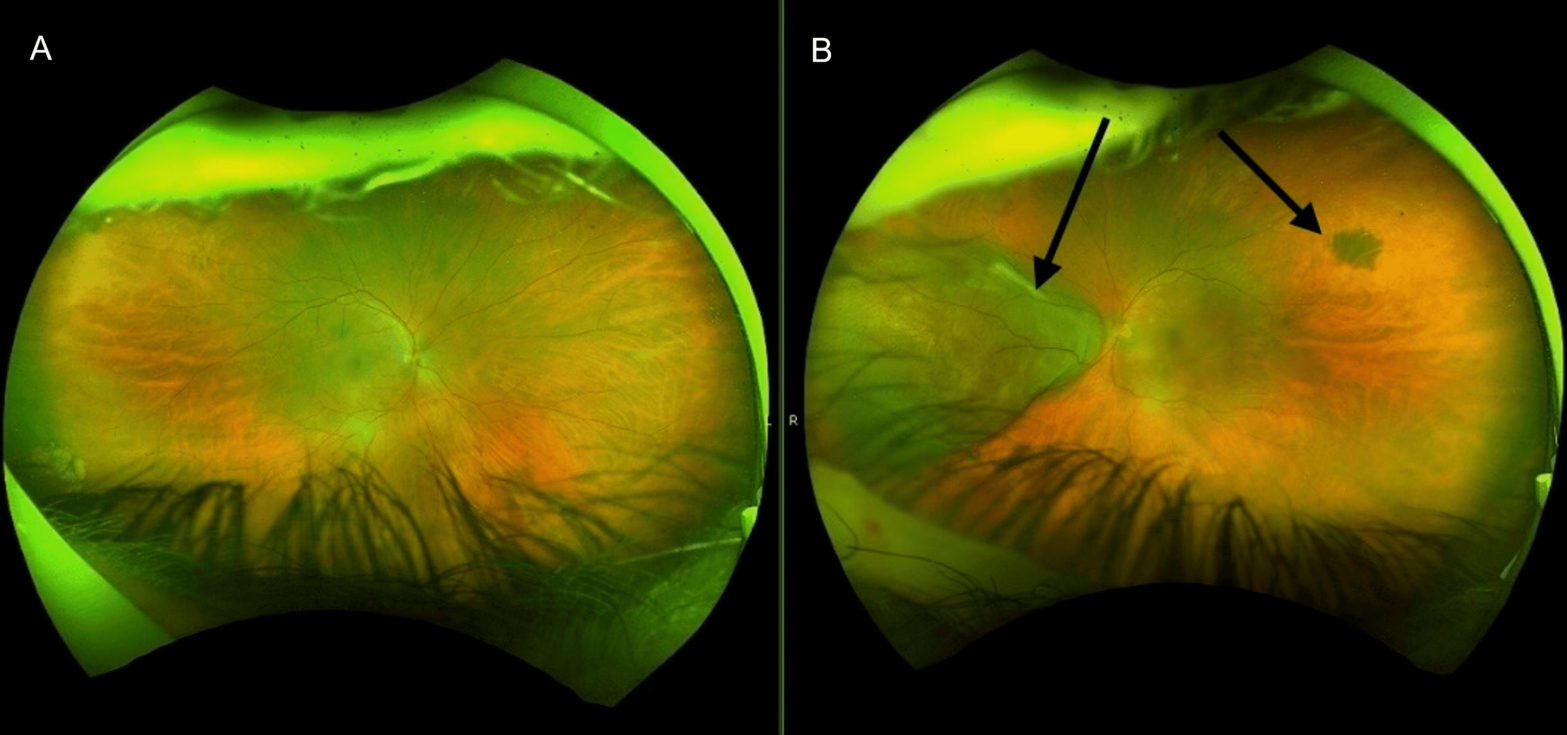
Color fundus photography of both eyes (A) Optos of the right eye showing a normal attached retina with no lesions. (B) Optos of the left eye showing an elevated mass nasally with overlying subretinal fluid and orange pigment (left arrow) and superotemporal small flat nevus (right arrow).

The patient was referred to ophthalmology for same-day evaluation. He was found to have an afferent pupillary defect of the left eye. The retinal examination was notable for a large choroidal pigmented elevated lesion nasal to the optic nerve head without surrounding heme or central drusen. There was a note of a possible retinal detachment and surrounding subretinal fluid (Figure 1). Bright scan ultrasonography demonstrated a 5.36 mm x 9.05 mm mass with low internal reflectivity. The differential diagnosis included choroidal melanoma, choroidal hemangioma, choroidal metastasis, choroidal nevus, or atypical choroidal neovascular membrane. There was high suspicion for choroidal melanoma given the mass’s thickness of greater than 2 mm, subretinal fluid with overlying exudative retinal detachment, margin within 3 mm of the optic nerve head, hollow ultrasound, and absence of central drusen.

Subsequent dilated fundus examination one week later showed a posterior choroidal melanoma in a classic dome shape measuring 11.5 mm x 16.5 mm with a maximum thickness of 5.2 mm. There was also a temporal flat nevus, which ophthalmology elected to observe. There were no signs of anterior or iris metastases on ultrasound biomicroscopy. 

The patient underwent transscleral choroidal sampling for gene expression profiling (GEP) and cryotherapy with a triple freeze-thaw cycle. Genetics confirmed class 1b uveal melanoma with PRAME positivity and mutations in GNAQ and SF3B1. CT of the chest/abdomen/pelvis, MRI of the abdomen with contrast, and PET/CT were obtained for staging purposes, all of which were negative for metastases. He was ultimately diagnosed with stage IIB (CT3aN0Mx) uveal melanoma. He received four sessions of urgent proton beam therapy with curative intent. He had near-immediate improvements in his visual fields following radiation therapy, likely due to tumor regression allowing for resorption of subretinal fluid and resolution of his retinal detachment. He was scheduled for close ophthalmology follow-up, as well as continued screening with MRI of the abdomen with contrast and CT of the chest with contrast every six months for the next 10 years. 

## Discussion

We describe a case of choroidal malignant melanoma, which was identified in the primary care setting during evaluation of superior quandrantanopia. Ocular melanoma remains a diagnostic challenge due to its low prevalence, vague presenting symptoms, and large phenotypic overlap with benign lesions [7]. Our patient’s only presenting symptom was a nonspecific black shadow in the superolateral visual quadrant of his left eye. CT of the brain raised concern for retinal detachment and prompted the need for urgent ophthalmologic evaluation. Choroidal melanoma most commonly presents with blurred vision, but can also cause visual field shadows, photopsia, floaters, and metamorphopsia. Of note, 13% of patients with choroidal melanoma are asymptomatic [8]. As in our patient, the visual symptoms in choroidal melanoma are largely attributed to concomitant exudative retinal detachments, which can occur in up to 75% of cases [9]. Larger tumors and those with a higher concentration of microvascular networks have more extensive retinal detachments and thus more severe visual deficits, but the presence of retinal detachments has not been shown to be an independent predictor of tumor aggressiveness [9]. 

Multimodal imaging, including fundus photography, optical coherence tomography, and ultrasound, can improve the detection rate of high-risk choroidal melanocytic lesions [10]. Multimodal imaging can also assist in identifying risk factors for malignant transformation, including lesion thickness >2 mm, presence of subretinal fluid, vision loss of 20/50 or worse per Snellen acuity, presence of orange pigment, acoustic hollowness, or lesion diameter >5 mm [11]. 

There have been several clinical, histologic, and cytogenetic features found to be predictive of poor prognosis; however, GEP has emerged as the superior option for determining metastatic potential and overall prognosis [12]. Based on a 15-gene quantitative PCR (qPCR)-based assay, tumors are classified into class 1a, 1b, or 2, which correspond to low, intermediate, and high risk of metastasis, respectively. More recently, the detection of PRAME mRNA has been used to further stratify metastatic risk in choroidal melanoma [13]. 

Plaque brachytherapy is the preferred treatment modality for localized choroidal melanoma due to its superior local control rates and lower incidence of treatment-related visual loss [14]. Despite excellent response rates to brachytherapy, the risk of recurrence is substantial, with studies showing a five-year recurrence rate of 17% [4]. Moreover, local recurrence has been found to be associated with a 6.28 times higher risk of systemic metastasis [15]. Thus, long-term surveillance with both medical oncology and ophthalmology is essential in the management of these patients. Surveillance typically focuses on imaging of the liver, the most common site of metastasis, with a frequency that depends on initial GEP classification and PRAME positivity. 

## Conclusions

Choroidal melanoma is a rare intraocular tumor that can present in the primary care setting with nonspecific visual symptoms. Accurate diagnosis requires a high index of suspicion, thorough physical examination, multimodal imaging, and prompt multidisciplinary involvement of ophthalmology and medical oncology. Early detection is essential, as treatment options in metastatic disease are limited and mortality rates are significant. As a primary care physician, findings of isolated visual field defects should prompt urgent fundoscopic evaluation. With timely identification and treatment of localized disease, outcomes are favorable. 

## References

[1] Singh AD, Topham A (2003). Incidence of uveal melanoma in the United States: 1973-1997. Ophthalmology.

[2] Fallico M, Raciti G, Longo A (2021). Current molecular and clinical insights into uveal melanoma (review). Int J Oncol.

[3] Ortega MA, Fraile-Martínez O, García-Honduvilla N, Coca S, Álvarez-Mon M, Buján J, Teus MA (2020). Update on uveal melanoma: translational research from biology to clinical practice (review). Int J Oncol.

[4] Jamison A, Bhatti LA, Sobti MM, Chadha V, Cauchi P, Kemp EG (2019). Uveal melanoma-associated survival in Scotland. Eye (Lond).

[5] Gallo B, Hussain R, Al-Jamal R (2024). Local tumour control and patient survival after ruthenium-106 brachytherapy for small choroidal melanoma. Br J Ophthalmol.

[6] Bellocq D, Roy P, Kodjikian L (2020). 20-year assessment of metastatic latency and subsequent time to death after proton therapy for uveal melanomas. Melanoma Res.

[7] Mishra K, Mruthyunjaya P (2025). The small choroidal melanoma: diagnosis, treatment outcomes, and unending controversy. Semin Ophthalmol.

[8] Sabazade S, Gill V, Herrspiegel C, Stålhammar G (2022). Vasculogenic mimicry correlates to presenting symptoms and mortality in uveal melanoma. J Cancer Res Clin Oncol.

[9] Kivelä T, Eskelin S, Mäkitie T, Summanen P (2001). Exudative retinal detachment from malignant uveal melanoma: predictors and prognostic significance. Invest Ophthalmol Vis Sci.

[10] Churchill RA, Pecoraro TY, Tooley AA, Houghton OM, Mashayekhi A, Dalvin LA (2024). Multimodal imaging risk factors predictive of small choroidal melanocytic lesion growth to melanoma: an educational study and pictorial guide. Eye (Lond).

[11] Shields CL, Dalvin LA, Ancona-Lezama D (2019). Choroidal nevus imaging features in 3,806 cases and risk factors for transformation into melanoma in 2,355 cases: the 2020 Taylor R. Smith and Victor T. Curtin lecture. Retina.

[12] Petrausch U, Martus P, Tönnies H (2008). Significance of gene expression analysis in uveal melanoma in comparison to standard risk factors for risk assessment of subsequent metastases. Eye (Lond).

[13] Harbour JW, Correa ZM, Schefler AC (2024). 15-gene expression profile and PRAME as integrated prognostic test for uveal melanoma: first report of Collaborative Ocular Oncology Group Study No. 2 (Coog2.1). J Clin Oncol.

[14] Bai H, Bosch JJ, Heindl LM (2023). Current management of uveal melanoma: a review. Clin Exp Ophthalmol.

[15] Gallie B, Simpson E, Saakyan S (2016). Local recurrence significantly increases the risk of metastatic uveal melanoma. Ophthalmology.

